# Looking back at 20 years of Open Access publishing at *Nucleic Acids Research*

**DOI:** 10.1093/nar/gkaf203

**Published:** 2025-03-26

**Authors:** Rich Roberts

When *Nucleic Acids Research* (NAR) was launched in 1974, the concept of an Open Access publication model was not on anyone’s radar. Instead, the focus was on how to ensure that the articles we published were of the very highest quality in the various fields of research involving nucleic acids. In the early 1990s, as the early internet became established, Paul Ginsparg founded an archive of papers in physics called the *arXiv* (https://info.arxiv.org/about/index.html) that became very popular (https://www.cs.cornell.edu/∼ginsparg/physics/blurb/pg01unesco.html). These papers were posted online and freely available to all. However, it was not until 2000 that related open access initiatives in biomedical sciences were launched, leading to the indexing service and full-text archive *PubMed Central* by NIH (https://pmc.ncbi.nlm.nih.gov), and the for-profit publisher *BioMed Central* by the then Current Science Group (https://sciencenow.com/aboutus.html), allowing biomedical researchers to freely access some selected published papers online.

In the early 2000s, many biological scientists began to seriously think about the most effective way for their research to be made available to everyone who wanted to read it. The Public Library of Science (PLOS) was formed in 2000 (https://en.wikipedia.org/wiki/PLOS), launching its first journal, *PLOS Biology*, in 2003 and we, at NAR, started to think about ways in which we could expedite access to the articles we published beyond the libraries that subscribed to the journal. At first, Oxford University Press was resistant to the idea of changing from the traditional subscription publishing model (NAR would become OUP’s first open access journal, so this was a major change). However, after a great deal of discussion, and strong advocacy for open access from the editors, OUP slowly began to consider the idea that NAR should become open access, and in 2005, it became a reality. We were one of the very first journals to embrace open access and it soon proved extremely popular with our authors and especially with our readers.

Since moving to an open access model, the popularity of NAR has increased over the years and the quality of the journal has steadily risen. Today, we are considered one of the very best journals in which to publish research findings in the molecular and cellular life sciences with a focus on nucleic acids. While much of our success can be attributed to the move to open access, it is still disappointing that many journals still refuse to be totally open access immediately upon publication and, in some cases, charge extraordinarily high fees to allow authors to present their work immediately for everyone to see. We had hoped that our move, as the first major traditional print journal to embrace open access, would encourage all journals to follow suit.

At the present time, it would appear that some publishers view scientific articles merely as an income stream and are reluctant to make sure that the discoveries coming from research, usually paid for by taxpayers and charitable foundations, are freely available to everyone both academics and the public. I sincerely hope that this will change soon.

